# Temperature-Dependent Thermal Properties of Nearly Amorphous Polyamide 6

**DOI:** 10.3390/polym18080981

**Published:** 2026-04-17

**Authors:** Julian Klingenbeck, Alexander Lion, Michael Johlitz

**Affiliations:** Institute for Mechanics, University of the Bundeswehr Munich, 85577 Neubiberg, Germany

**Keywords:** fused filament fabrication (FFF), FDM, PA6, nylon, heat capacity, thermal conductivity, density

## Abstract

The Fused Filament Fabrication (FFF) process has established itself as a key technology in prototyping and development and has garnered increasing interest in academic research. A substantial body of research on the FFF process has focused on the influence of process parameters on the resultant material/part properties. The thermal history of the printed part has proven itself as one of the most important factors in the printing process. It influences warping behavior, dimensional accuracy, build plate adhesion, as well as the mechanical properties of the finished part. A key requirement for understanding the influence of thermal history is the knowledge of the thermal properties of the considered material. In this study, the temperature-dependent thermal properties (isobaric heat capacity, thermal conductivity and density) of an unfilled polyamide 6 material for 3D printing are provided. Special attention is given to discussing the challenges associated with measuring these properties, particularly regarding how well the measured values represent the actual conditions during the printing process.

## 1. Introduction

The polyamide family of semi-crystalline polymers has been widely used in manufacturing for decades. While the first member of the polyamide family (polyamide 66) was commercialized for the production of synthetic fibers before 1940 [1], its proliferation and use cases expanded over the years. Nowadays, polyamide 6 is an engineering polymer primarily used in the automotive and electronics industries. Its ease of use allows processing it with all techniques common for thermoplastic polymers [2]. These same properties make it a desirable material for the Fused Filament Fabrication (FFF) process as well.

Fused Filament Fabrication denotes all those 3D printing processes in which a part is generated through melting of a prefabricated filament, which is then deposited in a line- and layer-wise fashion on a build plate [3]. The connection between the deposited polymer lines forms through a self-diffusion process, whereby polymer chains diffuse across the contact area of neighboring polymer lines forming a durable connection. Previous research has shown that the quality/completeness of this diffusion process has a significant impact on the resultant mechanical properties of the printed structure [4,5]. As the diffusion process itself is solely driven by the energy provided through the heat in the material, the thermal history during the printing process significantly impacts the mechanical properties of the printed parts, especially since cooling rates during the FFF process can reach several hundreds of degrees per second [6,7,8]. Further, the thermal history (or thermal gradients forming inside the part during the printing process) influences other quality and production related factors, such as warping, dimensional accuracy, and build plate adhesion.

It is therefore evident that, to assess the applicability of a material for 3D printing or potentially obtain the thermal history during the printing process via thermal simulations, the thermal properties of the material are required. The goal of this study is to provide the relevant thermal properties, i.e., heat capacity, thermal conductivity, and density, as functions of temperature for our chosen material, utilizing Differential Scanning Calorimetry (DSC), Hot Disk, Thermomechanical Analysis (TMA) and pycnometry. The material investigated is an unfilled polyamide 6 filament (F3 Pa Pure Pro) manufactured by Fiberthree. More specifically, the thermal properties presented in this study are intended to represent the behavior of polyamide 6 under FFF printing conditions. To obtain such representative results, special care must be given to the influence of the sample manufacturing and evaluation methods, as it will be discussed below.

### 1.1. Preliminary Considerations

Before an appropriate experimental methodology can be established some important topics have to be discussed. Firstly, the requirements of the different testing methods on sample preparation and unwanted influences of the measurement procedures on the measured thermal properties and how to handle them in the context of data preparation. Secondly, the moisture content and the crystallinity of the material. Both influence the mechanical and thermal properties of semi-crystalline thermoplastics.

#### 1.1.1. Moisture Content

Thermoplastic polymers and polyamides especially are prone to taking up moisture from surrounding media or air. Increased moisture content leads to a reduction in mechanical parameters—such as Young’s Modulus, Hardness, etc.—and changes in the thermal properties [2]. In the context of FFF printing, the material is fully molten during the production process. With a melting temperature above 200 °C, we assume that no moisture is present in the material during and directly after the printing process. As it is intended for the measurements in this study to represent the material during the production process, it is necessary to maintain a moisture content as low as possible.

#### 1.1.2. Crystallinity

As the name semi-crystalline thermoplastic polymer implies, such materials are known to form crystalline structures during the cooling process. In the context of polymers, this refers to the material’s ability to form a long-range order with the crystal lattice type depending on the polymer in question. These crystalline structures further organize in super-molecular structures, such as lamellae, spherulites, and more. Semi-crystalline polymers never reach a state of complete crystallinity. A significant portion of the polymer remains amorphous, with only short-range order present [2,9,10]. The amorphous and crystalline phases of polyamide 6 possess distinct mechanical and thermal properties. Thus, the overall properties of the polymer are dependent on the proportion of the crystalline and amorphous phase, expressed commonly by the degree of crystallinity. Higher crystallinity is correlated with higher values in the mechanical parameters as well as higher thermal conductivity and lower heat capacity. The formation of the crystalline phase takes place during the cooling process and is highly dependent on the cooling rates experienced by the material. Care must therefore be taken to ensure that the degree of crystallinity in any tested samples is comparable to those developed during the FFF process.

## 2. Methodology

In the following section, the principles of operation of all employed measurement methods are briefly summarized, followed by the measurement parameters employed and some additional comments.

### 2.1. Sample Preparation

To recall, the thermal properties intended to be measured in this study are the heat capacity, the thermal conductivity, and the density. Heat capacity in form of the isobaric heat capacity as well as the degree of crystallinity are determined via Differential Scanning Calorimetry (DSC). The thermal conductivity is established with the Hot Disk method. For the temperature-dependent density, a combination of Thermomechanical Analysis (TMA) and Pycnometer experiments are used.

For TMA, Hot Disk and Pycnometer measurements, samples with minimal porosity or voids are required to deliver high-quality results. TMA and Hot Disk further require samples with at least two smooth parallel surfaces. The DSC method does not share the same stringent geometry requirements and can tolerate a moderate surface roughness. To meet the set requirements samples of the identical base material (F3 Pa Pure Pro by Fiberthree) were produced via both injection molding and 3D printing.

For all tests (further described in Section 2), appropriately sized samples were cut from injection-molded tensile specimen (Type 1A DIN 527-2 [11]). Injection molding ensures that the samples are free of porosity and provide smooth surfaces. Three-dimensional-printed samples were only used for the DSC tests to determine whether comparability between printed and injection molded samples can be assumed. The samples were cut from printed boxes (50 mm × 50 mm × 50 mm) with a wall thickness of a singular polymer line (width: 0.4 mm, height: 0.2 mm), no infill, and no top/bottom layers. A German RepRap X500 (InnovatiQ GmbH (formerly: German RepRap), Feldkirchen, Germany) was used for the printing process, with an extrusion temperature of 285 °C, bed temperature of 60 °C and 50 mm/s printing speed under ambient temperature. These samples ensure, as with the injection-molded ones, that the material is free of porosity. They exhibit, however, the characteristically uneven surface of FFF-printed parts, which makes them unsuitable for TMA and too thin for Hot Disk measurements. For DSC measurements, this is no drawback.

**Figure 1:**
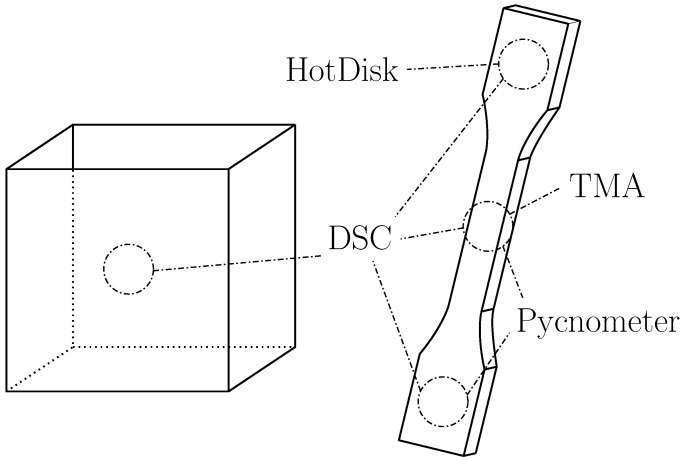
Extraction positions for injection molded and 3D-printed samples.

Since the reported cooling speeds in the FFF process suggest that the resultant material may be nearly amorphous, it was chosen to include a third sample type in the form of ice-quenched material in the DSC runs to represent the most amorphous form of the material within the scope of this study. The quenched samples were produced with the German RepRap X500 printer by extruding the material from the 285 °C nozzle directly into an ice water bath. Figure 1 shows the extraction positions for the different testing methods.

To avoid any influence of moisture content during subsequent testing, all samples utilized in this study were dried in a convection oven (Nabertherm TR60, Nabertherm GmbH, Lilienthal, Germany) at 70 °C for 24 h according to the recommendations of the material manufacturer and subsequently stored in a dry cabinet (MP Dry Cabinet IV ST, MP Elektronik Technologie, Svitávka, The Czech Republic) at 35 °C and relative humidity below 1% RH until testing.

### 2.2. Data Interpretation

For all measurement methods employed in this study, with the exception of the Pycnometer, the sample is heated across a wide temperature range to obtain the temperature-dependent material data. Unfortunately, if a semi-crystalline polymer is frozen in an amorphous or near-amorphous state due to fast cooling rates (as they are present during FFF printing of thin structures), cold crystallization may occur during the heating process. As the polymer is heated above its glass transition temperature, some of the amorphous phase regains enough mobility to form new crystalline structures, thereby changing the sample’s composition during the measurement process. Therefore, the measured material parameters do not represent the material state as it would be present during the printing process. As it was further impossible to replicate the fast cooling rates of the FFF process (in the range of several hundred degrees per second) with the available testing methods, the material properties could not be measured during cooling directly. For that reason, it was chosen to derive synthetic material curves from the measured results. The specific ways of obtaining the synthetic data vary between the tested properties and are elaborated upon in the appropriate sections. All data analyses were conducted using custom Python scripts (version 3.12.1), employing the packages NumPy (version 1.26.3), pandas (version 2.1.4), SciPy (version 1.11.4), and Matplotlib (version 3.8.2).

### 2.3. Differential Scanning Calorimetry

All DSC measurements presented in this study were performed on a TA Instruments Q1000 DSC (Waters Corporation, New Castle, DE, USA), calibrated for heat capacity measurements with a heat–cool–heat protocol between 0 and 260 °C at a constant heating and cooling rate of 10 °/min, a Nitrogen purge gas flow rate of 50 mL/min, and isothermal holding times of 5 min at the end of each heating and cooling ramp (based on recommendations from DIN EN ISO 11357-3) [10,12,13,14,15,16,17]. Three samples, with a mass of between 3 and 5 mg per sample type, were cut from the material for a total of five groups. Injection-molded samples were extracted from three positions of the tensile specimen: the gauge region (denoted in the following as IM Gauge), the clamping region close to the injection point (IM Near), and the clamping region far from the injection point (IM Far). The 3D-printed samples were extracted from a central position in the walls of the printed box. The quenchd samples were obtained by cutting appropriately sized samples from the quenched extrudate.

The results were evaluated, firstly, with regard to the degree of crystallinity, comparing the injection molded samples to the printed samples. The calculation of the degree of crystallinity has been the topic of continuous research. Previous research suggests that the results measured with the standard evaluation method are overestimated compared to results obtained from other techniques, such as X-ray diffraction. Especially for near-amorphous materials, this may result in higher values of absolute crystallinity than other methods would suggest. It should, however, be noted that the DSC method provides comparable accuracy to XRD for identifying relative changes in crystallinity between samples of the same material, which is the primary purpose of the crystallinity measurements in this study [18,19]. Nonetheless, to correct for this circumstance, a variety of alternative or improved evaluation methods have been proposed in literature. For the purpose of this study, three evaluation methods were compared: the standard method [16], a method used by Millot et al. [20], and a method proposed by Khanna et al. [21]. Further explications of these methods are provided below.

Following this, the measured heat capacity curve was used to generate a synthetic heat capacity curve intended to reflect the material property during the printing process.

### 2.4. Hot Disk

The thermal conductivity measurements presented in this study were performed on a Hot Disk TPS 2500s and a Kapton 7577 sensor (Hot Disk AB, Göteborg, Sweden). Disk-shaped samples of approx. 20 mm in diameter and 4 mm in thickness were cut from the clamping section of injection molded tensile specimens. A heating power of 15 mW and a measurement time of 20 s were determined in preliminary tests to be suitable for the given material and sample dimensions. The measurement setup (sample, sensor, sample holder) was placed in a Nabertherm TR 120 oven to allow for measurements at varying temperatures. The thermal conductivity of the material was determined at several temperatures between room temperature and 140 °C, with 5 repetitions per temperature level. The material was allowed to reach equilibrium at the defined temperature levels after which an isothermal period was maintained for at least 10 min prior to testing. A total of two heating cycles were performed [16,22,23].

Subsequently, the synthetic thermal conductivity curve was created.

### 2.5. Thermomechanical Analysis and Pycnometer

Combining a reference density measured via Pycnometer at a given temperature with the linear expansion measured via TMA, the temperature-dependent density can be approximated according to the below formula, which follows from the conservation of mass under the assumption of an isotropic material:

(1) $$\rho \left(\theta \right)=\frac{\rho_{0}}{1+3\frac{\Delta L(\theta )}{L_{0}}}$$

The reference density is denoted as $\rho_{0}$, the temperature-dependent change in sample length as ΔL(θ), and the initial length is described by $L_{0}$.

TMA measurements were performed on a Mettler Toledo TMA/SDTA 841e (Mettler-Toledo International Inc., Columbus, OH, USA) on cubic samples (4 mm × 4 mm × 4 mm), cut from injection molded tensile specimen, with a preload of 0.1 Newton between 0 °C and 140 °C for 6 samples. A total of 3 heating–cooling cycles between the defined temperature boundaries were performed at a constant heating and cooling rate of 5 °C/min, with 5 min isothermal periods at the end of each heating and cooling ramp. Pycnometer measurements were performed on an Anton Paar Ultrapyc 5000 (Anton Paar GmbH, Graz, Austria) at 20 °C on injection molded samples [10,16,24,25,26]. As before, a synthetic density curve was constructed from the measured values and extrapolated to the full temperature range.

## 3. Results and Discussion

### 3.1. DSC—Results

#### 3.1.1. Crystallinity

**Figure 2:**
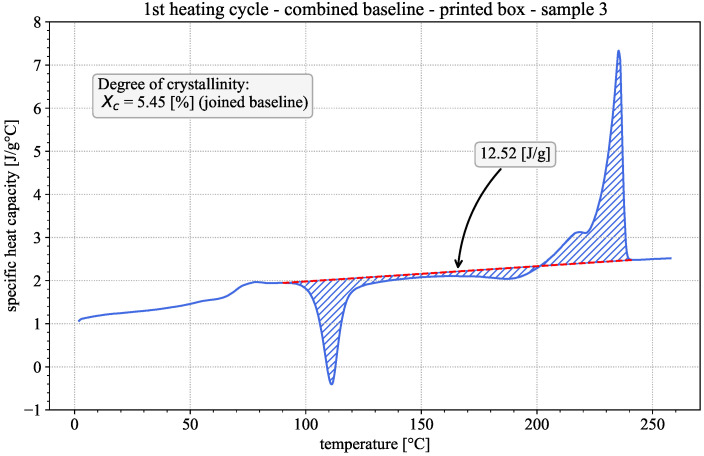
Apparent heat capacity in the first DSC heating cycle of a printed sample, including a combined baseline (dashed red line), enthalpy of crystallization (hatched blue area) and crystallinity evaluation, according to Khanna et al. [21].

**Figure 3:**
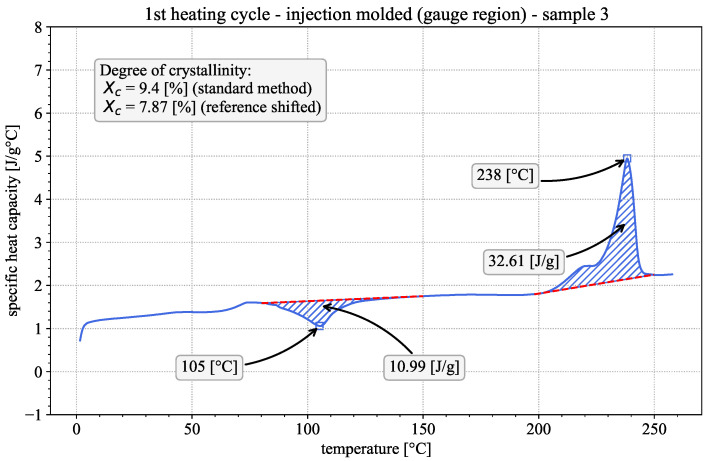
Apparent heat capacity in the first DSC heating cycle of an injection-molded sample, including separate baselines (dashed red lines), enthalpy of crystallization (hatched blue area) and crystallinity evaluation, according to the standard method and the method by Millot et al. [20].

First insights into the material properties and the influence of the printing process can be gained by qualitatively evaluating the DSC results of the 1st heating cycles of both the printed and the injection-molded samples. Representative examples of both are given in Figure 2 and Figure 3. The observed behavior is typical for semi-crystalline thermoplastic polymers [16,17]. In both cases, the apparent heat capacity varies with temperature, showing three distinct regions of interest. The step-like glass-transition region is at approx. 75 °C, followed by an exothermic peak at approx. 110 °C, indicative of the occurrence of cold crystallization. The endothermic melting peak is at approx. 235.5 °C, preceded by a pronounced shoulder. The existence of the exothermic cold crystallization peak during the 1st heating suggests that the material was present in a near-amorphous state prior to testing [27]. Considering the fast cooling rates during the FFF printing process, this appears to be a reasonable assumption.

During cold crystallization, parts of the previously amorphous phase rearrange and form new crystalline phases. Polyamide 6 is known to form three distinct crystalline phases denoted as α, β and γ phases. Without considering any chemical post-production treatments, none of these crystalline structures appear exclusively in polyamide 6 samples. Depending on the specific temperature history, one of the phases may be predominantly present. If the samples are indeed close to totally amorphous prior to DSC testing, the slow heating rates during the test should, according to Seguela [27], predominantly result in the development of the mesostable β phase. The shoulder prior to the melting peak may be attributed either to the existence of a second crystalline phase or further rearrangement in the crystalline structure before melting occurs. For the purposes of this study, the exact proportion of the individual crystalline phases is not relevant. For further information, the reader can refer to the literature [21,27,28,29,30,31].

Since the printed and the injection-molded specimens show cold crystallization and a qualitatively similar behavior, it may be assumed that in both manufacturing processes the cooling rates are fast enough to prevent major crystallization. To quantify this observation, the degree of crystallinity was calculated following the three methods mentioned in Section 2.3.

The standard evaluation method is to draw linear baselines above/below the cold-crystallization and melting peaks as can be seen in Figure 3. Subsequently, the areas enclosed between the peaks and their respective baselines are calculated. The calculated areas represent the released/absorbed enthalpy in [J/g] of the material during cold crystallization and melting, which are used to calculate the degree of crystallinity according to [14,16,17]:

(2) $$\chi_{c}=\frac{H_{m}-H_{c}}{H_{f}^{0}}$$

The melting and cold crystallization enthalpies are denoted as $H_{m}$ and $H_{c}$, respectively, with the reference enthalpy $H_{f}^{0}$ being the melting enthalpy of the 100% crystalline material, taken from literature to be 230 J/g [10] for PA6. However, with this evaluation method, a problem arises. Since the crystalline phase of the material requires a higher melting enthalpy than the amorphous phase, the melting peak is shifted to higher temperatures for higher degrees of crystallinity. The reference enthalpy used above was measured at approx. 259.85 °C (533 K). Considering the substantial temperature range between the reference and the experimental melting and cold-crystallization peaks, it has been suggested in the literature [20,32] that the temperature-dependence of the reference enthalpy should be corrected for. This is due to the fact that the variation in the heat capacities over temperature differs for the liquid (amorphous) and solid (crystalline) phases. The evaluation below follows the example of Millot et al. [20] utilizing Kirchhoff’s law, which can be used to shift the reaction enthalpy of any chemical reaction (including phase changes) to any temperature if the change in heat capacity of the reacting mixture is known.

(3) $$\Delta H_{f}\left(T_{2}\right)-\Delta H_{f}\left(T_{1}\right)=\int \Delta c_{p}\;dT$$

$\Delta H_{f}\left(T_{2}\right)$ and $\Delta H_{f}\left(T_{1}\right)$ represent the reaction enthalpies at two temperatures, and $\Delta c_{p}$ denotes the change in heat capacity due to the reaction. Since this shift is applied to the reference enthalpy, $\Delta c_{p}$ refers to the difference between the heat capacity of the liquid and the solid polymer.

(4) $$\Delta c_{p}=c_{p,l}\left(T\right)-c_{p,s}\left(T\right)$$

Using the heat capacity data provided by Gauer et al. [33], the reference enthalpy (230 J/g at 533 K) can be shifted to the measured temperature peaks for cold crystallization and melting. The degree of crystallinity is then calculated as:

(5) $$\chi_{c}=\frac{H_{m}}{H_{f}^{0}\left(\theta_{m}\right)}-\frac{H_{c}}{H_{f}^{0}\left(\theta_{c}\right)}$$

with two separate reference enthalpies associated with the peak temperatures of cold crystallization and melting, respectively. Detailed calculations for the reference enthalpy are provided in the Supplementary Materials.

A third evaluation method is presented by Khanna et al. [21]. They note that, specifically for PA6, changes in crystallinity can occur between the pronounced cold crystallization and melting regions, which are not accounted for if two separate baselines are used. They therefore suggest the use of a combined linear baseline from a point before cold crystallization up to a point after melting, as depicted in Figure 2. The start and end points of the combined baseline can be chosen to be the same as the start of the cold crystallization baseline and end of the melting baseline in the standard evaluation method. This leads to the calculation of the degree of crystallinity as:

(6) $$\chi_{c}=\frac{H_{c}omb}{H_{f}^{0}}$$

It should be noted that Khanna et al. [21] only recommend this method for PA6 with low crystallinity (below 25%), which is the case in this study.

**Figure 4:**
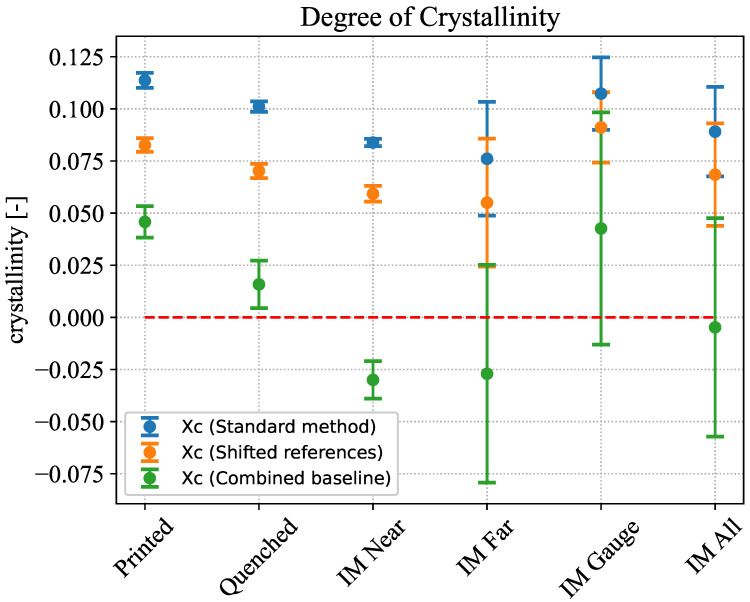
Crystallinity evaluation of all tested sample groups following the three evaluation methods with zero crystallinity highlighted in red.

Figure 4 shows the results of the crystallinity evaluations for all sample groups. Additionally the three injection molded sample groups were evaluated as a combined group (IM All). Table 1 additionally shows the mean values and standard deviations of cold crystallization temperature, melting temperature, and crystallinities of all tested groups.

For the standard evaluation method, crystallinities vary between 7.5% and 12%. The injection molded samples exhibit greater standard deviations than the printed one. However, considering that the mean values of all tested groups fall within the standard deviation of the injection-molded samples (IM All), it appears reasonable to assume all samples to be of comparable crystallinity. The evaluation following Millot et al. leads to a shift to lower crystallinities for all groups with values ranging between 5% and 10%. The evaluation according to Khanna et al. leads to the most drastic changes in crystallinity, pushing the value for all groups below 5%. The results, however, also show a substantial weakness of this method. When using a combined baseline, it is possible to obtain negative values for the enthalpy, leading consequently to negative crystallinity when measuring samples close to the amorphous state. This behavior is of course physically implausible and indicates a systematic limitation of the evaluation method. Since the baselines used to determine the cold crystallization and melting enthalpies are selected by the user, even minor variations in the chosen temperature ranges, as well as small measurement errors, can result in negative values, particularly for materials close to the amorphous state.

**Figure 5:**
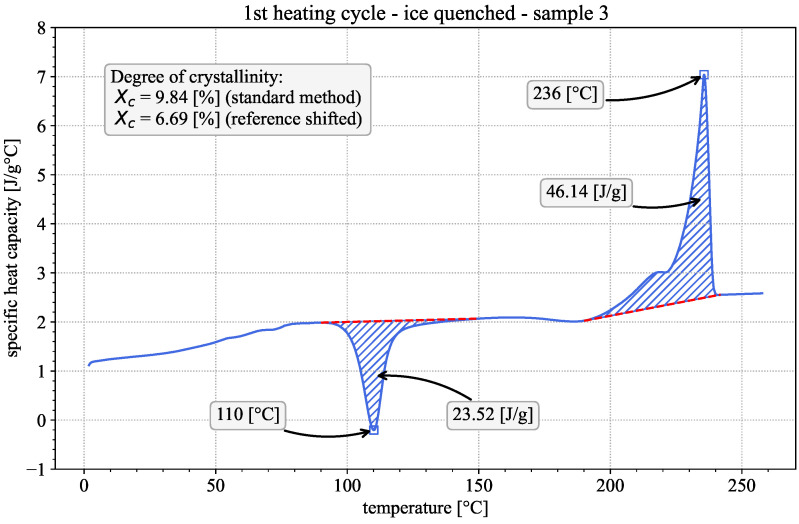
Apparent heat capacity in the first DSC heating cycle of an ice-quenched sample including seperate baselines (dashed red line), enthalpy of crystallization (hatched blue area) and crystallinity evaluation according to the standard method and the method by Millot et al. [20].

Based on these results it can be concluded that the printed and injection molded samples exhibit comparable degrees of crystallinity and thus it can be assumed that the thermal properties measured with injection-molded samples are comparable to those of the printed samples as well. Another important observation is the low crystallinity of the material in all cases. This becomes especially apparent when considering the results of the ice quenched samples, which can be assumed to have been subjected to the fastest cooling rates among the tested groups. Both the crystallinity results in Table 1 and the qualitative behavior of the heat capacity curve depicted in Figure 5 show a remarkable similarity between the printed and quenched samples. This suggests that the thin printed structures utilized in this study already possess little to no crystallinity. Given the difficulty in evaluating near-amorphous samples via DSC, it may reasonably be assumed that the material is entirely amorphous. This assumption is further corroborated by the fast cooling rates present in the FFF printing process, which hinder, if not prevent, the formation of crystalline phases during the cooling process. All reasoning regarding the construction of the synthetic data curves from the measured results discussed in the following sections will be based on this assumption.

#### 3.1.2. Heat Capacity

**Figure 6:**
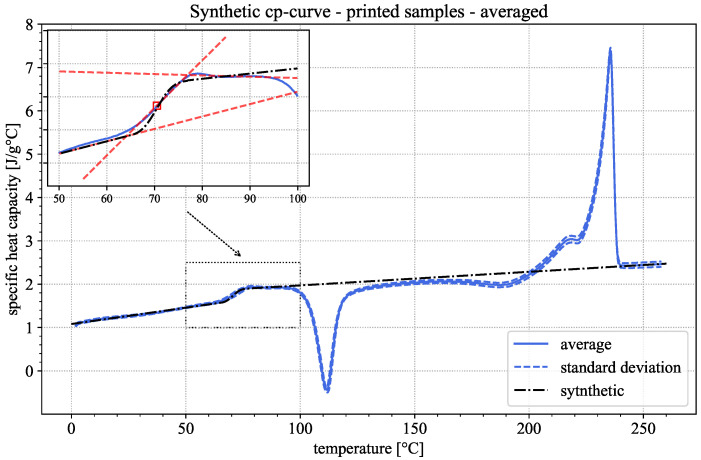
Averaged apparent heat capacity of the first DSC heating cycle (blue) and synthetic isobaric heat capacity curve (black) for 3D-printed samples according to Equations (7) and (8). The Zoom-In depicts the glass transition region of the material, indicating $T_{g}$ (red square mark) as well as the Onset- and Endset-temperatures (crossing points of the dashed red lines).

The heat capacity curve measured in a DSC experiment is referred to as the apparent heat capacity. This is due to the fact that the isobaric heat capacity is defined for a constant amount of substances in the tested material. In the test performed in this study, the amounts of crystalline and amorphous phases change during cold crystallization and melting. The measured apparent heat capacity curve is thus not representative of the material in its initial state. Under the assumption that the material is entirely amorphous, a synthetic heat capacity curve can, however, be constructed. To this end, the apparent heat capacity curve can be separated into three distinct regions: the region before glass transition occurs (pre $T_{g}$ region), which can be approximated linearly; the glass transition region ($T_{g}$ region), approximated via a Gaussian error function; and the region above glass transition (post $T_{g}$ region), which can be approximated linearly as well. For the fitting of the post $T_{g}$ region, the data points after glass transition, but before cold crystallization, and data points after the melting peak (i.e., the melt) are used. As the intention of this study is to find thermal properties representative of the material as it occurs during the printing process, the DSC-averaged results of the printed samples were used to construct the synthetic heat capacity curve depicted in Figure 6.

As stated above, the synthetic heat capacity was constructed over three distinct regions, with the following equations and constants depicted in Table 2 using the averaged DSC curves of all measurement repetitions for the FFF-printed samples.

(7) $$c_{p}\left(\theta \right)=\left\{\begin{array}{cc} m_{1}\theta +t_{1} & \mathrm{if}\;\theta <T_{g}\;\mathrm{Onset}\\ a+b*\frac{1}{2}\left(1+erf\left(\frac{\theta -c}{d}\right)\right) & \mathrm{if}\;T_{g}\;\mathrm{Onset}\leq \theta <T_{g}\;\mathrm{Endset}\\ m_{2}\theta +t_{2} & \mathrm{if}\;\theta \geq T_{g}\;\mathrm{Endset} \end{array}\right.$$

(8) $$erf\left(z\right)=\frac{2}{\sqrt{\pi }}\int_{0}^{z}e^{-t^{2}}\;dt$$

This leads to a relative change in the heat capacity between 20 °C and 260 °C of approx. 100% relative to the 20 °C value.

### 3.2. Hot Disk—Results

**Figure 7:**
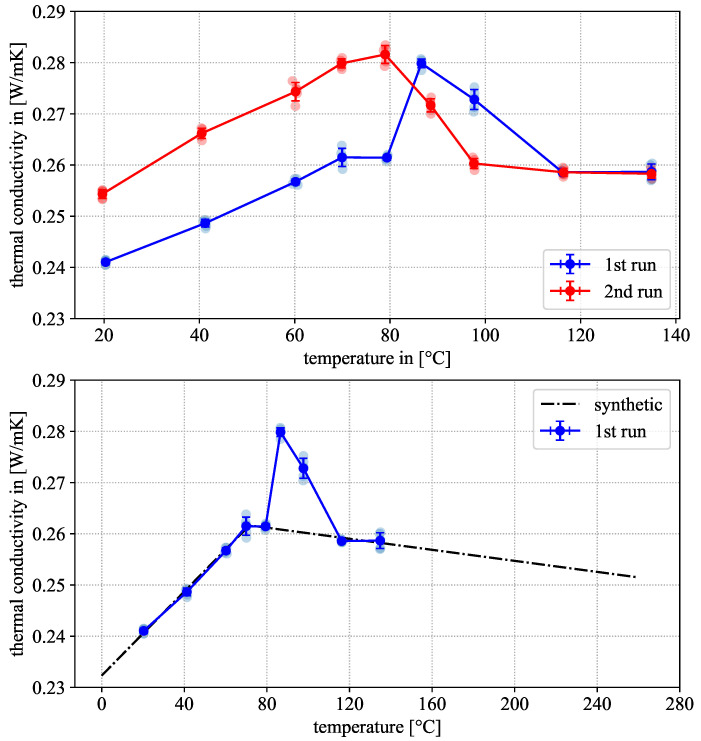
Thermal conductivity measured via Hot Disk in 1st (blue) and 2nd (red) heating run (**top**). Thermal conductivity in the 1st (blue) heating cycle and synthetic thermal conductivity curve based on Equation (9) (**bottom**). (Averages with standard deviation are shown in solid color, individual data points are displayed in a semi-transparent color).

The results of the thermal conductivity measurements are displayed in Figure 7. Depicted are two heating runs performed on the same sample. In the 1st heating run, the apparent thermal conductivity rises with increasing temperature until it levels off around 70 °C. During further heating, a peak occurs between 80 °C and 115 °C before the signal drops back to slightly below the plateau value above 120 °C. The reported temperature-dependent behavior for semi-crystalline polymers varies between materials, including rising, declining and constant thermal conductivity with temperature [16,34]. The measured behavior, an initial near-linear rise followed by a plateau or even slight decline at glass-transition temperature, has been reported similarly for PA6 [35]. The transition point between the linear rise and plateau regions correlates well with the glass-transition temperature via DSC, given the sparsity of data points available from the Hot Disk measurements. The peak in the plateau may be associated with cold crystallization taking place in this temperature region. This assumption is supported again by the DSC results, which show cold crystallization occurring in the same temperature region. The measurements are not directly comparable, as the DSC measurements took place under constant heating, while the Hot Disk measurements were performed at isothermal intervals. The temperature regions for cold crystallization might therefore be shifted between the experiments and should not be interpreted unequivocally.

**Figure 8:**
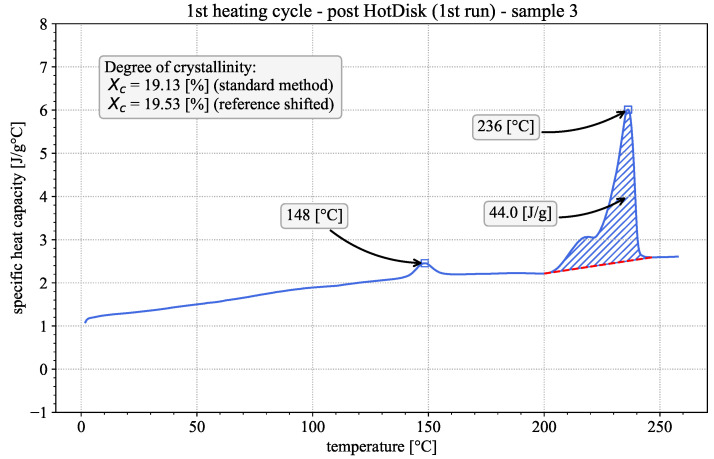
Apparent heat capacity in the first DSC heating cycle of a Hot Disk sample after the 1st Hot Disk heating run, including baseline (dashed red line), enthalpy of crystallization (hatched blue area) and crystallinity measurements.

**Figure 9:**
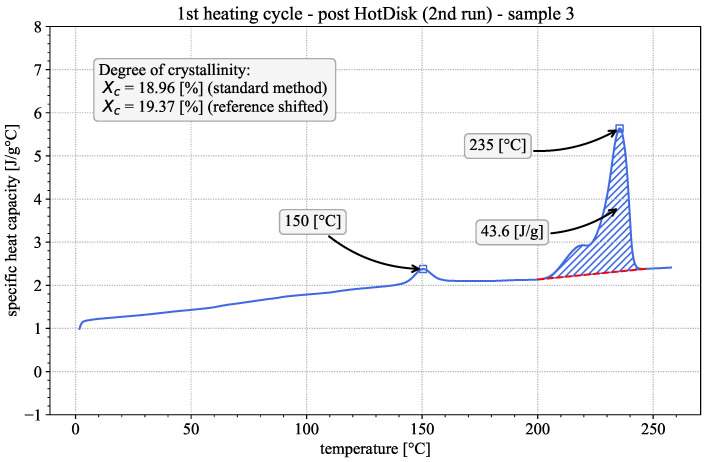
Apparent heat capacity in the first DSC heating cycle of a Hot Disk sample after the 2nd heating run, including baseline (dashed red line), enthalpy of crystallization (hatched blue area) and crystallinity measurements.

In the second heating run, prior to glass transition, thermal conductivity follows the same near-linear trend as before, shifted upwards by approx. 0.015 W/mK. This is consistent with theory, as the crystalline phases of polymers typically possess higher conductivity than their amorphous counterparts [36,37], which confirms that cold crystallization took place during the 1st heating run. Above 80 °C, thermal conductivity falls again until it stabilizes at the same plateau value as in the 1st heating cycle. The increase in crystallinity between the initial state and the 1st and 2nd heating runs is confirmed by additional DSC measurements depicted exemplarily in Figure 8 and Figure 9, performed on the sample after the respective heating cycles following the same methodology described in Section 2.3. Evaluation following the standard method showed crystallinities of 17.97±1.65% and 18.27±0.63% after the 1st and 2nd heating cycles, respectively. This represents a sizable increase compared to the initial material, as described in Section 3.1.1, although no significant difference in crystallinity could be established between the two post-measurement results.

One observation of note is the presence of an annealing peak at approx. 150 °C. These peaks typically only appear in a DSC measurement if the sample has been annealed at a constant temperature above the glass transition prior to testing [17]. The exact shape and position of the annealing peak depends both on annealing temperature and time. As the Hot Disk measurements were taken at stable temperature levels after an additional isothermal holding period, the sample was effectively annealed repeatedly at varying temperature levels. The presence of an annealing peak is therefore to be expected. Of further note is the absence of any cold crystallization peaks in the DSC curves. This is a further indication that the material has reached significantly higher levels of crystallinity post-Hot Disk measurement. Based on these observations, the construction of a synthetic thermal conductivity curve is based on the following assumptions. As it is intended to obtain data for a nearly amorphous material (as before with the DSC results), data from the 1st heating run were used prior to glass transition. At high temperature levels, no data for the amorphous material is available, as the material undergoes cold crystallization during heating and an extrapolation from the melt is not possible, as the measurement setup can only withstand temperatures up to 160 °C. Corroborated by the literature knowledge about thermal conductivity of thermoplastic polymers, we decided to approximate the thermal conductivity of the amorphous material by fitting a piece-wise linear function to the 1st heating cycle, while excluding the 90 °C and 100 °C temperature levels, as they are associated with the cold crystallization of the material. The resulting constructed synthetic thermal conductivity curve was further extrapolated above the material’s melting point and is given by the following equation and parameters in Table 3 and depicted by the dashed line in Figure 7.

(9) λ(θ)=aθ+bmax(0,(θ−c))+d

This leads to a relative change in thermal conductivity between 20 °C and 260 °C of approx. 4.6% relative to the 20 °C value.

### 3.3. TMA and Pyknometer—Results

**Figure 10:**
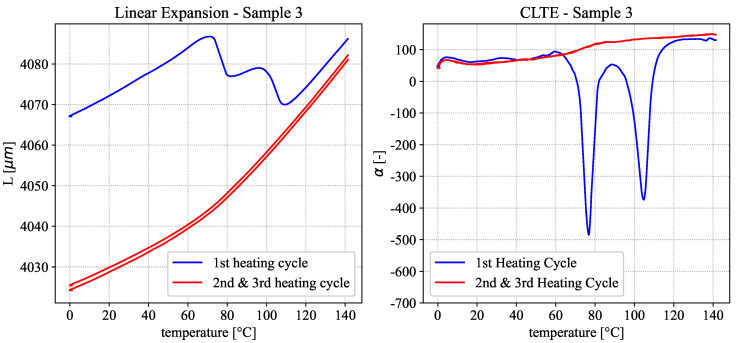
Thermal expansion of an injection-molded sample in three subsequent heating cycles (**left**). CTLE of the injection-molded sample in three subsequent heating cycles (**right**).

The initial data measured in TMA tests is the extension of a material given for a representative sample in Figure 10 (left) as the total length of the material over temperature. Depicted are the three heating cycles of the measurement protocol. As with the previous experiments, a clear difference between the 1st and the subsequent heating cycles is observed. During the 1st heating, the material expands in a near-linear fashion until approx. 70 °C, when a sudden step-like drop occurs, which can be correlated with the glass transition of the material. This sudden shrinkage may be due to a release of internal stresses present in the material prior to measurement caused by the increased mobility of the polymer chains above the glass transition temperature. A second drop occurs around 100 °C, which is correlated with cold crystallization. Since the crystalline phase is typically denser than the amorphous one, a crystallization process consequently leads to a reduction in volume as was observed here. After cold crystallization, the curve rises again in a near-linear fashion with temperature. Both of these shrinkage effects are well documented for thermoplastic polymers [16].

In the subsequent heating cycles, these step-like drops are no longer present since all possible relaxation and cold crystallization effects have been completed for the given temperature profile. The total length of the material remains consistently below that of the first heating cycle, following a piece-wise linear behavior with a bend at the glass transition, where the steepness of the curve changes. Considering the Coefficient of Linear Thermal Expansion (CLTE) depicted in Figure 10 (right), we can further establish that the CLTE remains identical for all measurement runs outside of the phase transition regions. As the CLTE is simply the derivative of thermal expansion with respect to temperature, this means that the near-linear sections of the absolute length curve have the same slopes across all heating runs. To arrive at a simple synthetic data curve of the temperature-dependent density, the shrinkage at the glass transition and cold-crystallization regions may be ignored. The former can be ignored because potential internal stresses are dependent on the thermal history the sample experienced and will be different depending on the manufacturing process and geometry. For the latter, the cold-crystallization only occurs during heating and does not represent the material’s actual structure during cooling. This effectively means that even for the nearly amorphous material, the 3rd heating cycle can be used as the basis for calculation without incurring too large an error.

**Figure 11:**
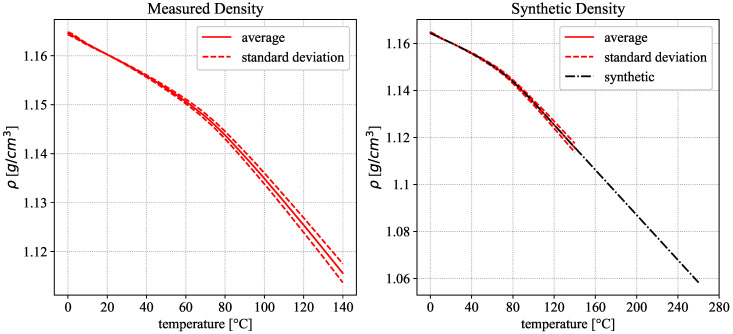
Density according to Equation (1) based on averaged values for thermal expansion and reference density (**left**). Measured density (red) and extrapolated synthetic density curve (black) according to Equation (10) (**right**).

Together with a reference density, measured with the Pycnometer to 1.1602 $g/{\mathrm{cm}}^{3}$ at 20 °C, the temperature-dependent density curve was calculated from the averaged 3rd heating cycle for all measured samples, according to the equation from Section 2.5, and is depicted in Figure 11 (left).

A synthetic density curve was established in two parts. The first part represents the density for the measured temperature range. The second part is a linear extrapolation to the higher temperature levels. The complete synthetic density curve can be described by the following equation and parameters in Table 4 and is depicted in Figure 11 (right).

(10) $$\rho \left(\theta \right)=\left\{\begin{array}{cc} a\;\theta +\left(b-a\right)\;\theta \frac{1}{1+exp(-c(\theta -d))}+e & \mathrm{if}\;\theta <140\;{}^{\circ}C\\ m\theta +t & \mathrm{if}\;\theta \geq 140\;{}^{\circ}C \end{array}\right.$$

This leads to a relative change in density between 20 °C and 260 °C of approx. 8.8% relative to the 20 °C value.

## 4. Conclusions

The thermal properties of a material are essential for understanding and potentially predicting the thermal history during the Fused Filament Fabrication process. In this study, the relevant properties for a thermal simulation (heat capacity, thermal conductivity and density) were established for the PA6 material Fiberthree F3 Pa Pure Pro. The results clearly show that the isobaric heat capacity exhibits the greatest change with increasing temperature in a range relevant for the FFF process (20–260 °C), with an increase of approx. 100%. The thermal conductivity and density vary far less with temperature, with relative changes of only 4.6% and 8.8%, respectively, over the same temperature range. For the purposes of thermal simulations, especially over large temperature ranges, the temperature-dependence of these parameters should not be ignored.

A major challenge in establishing these thermal properties for semi-crystalline polymers is to obtain a plausible estimation for the specific process or circumstances to be examined. Since the thermal properties are dependent on micro-structure, i.e., the amount of amorphous and crystalline phases present in the material, special attention must be given to how well the measured data reflect the actual material properties. In this study, we have made the plausible assumption that for the case of thin FFF-printed structures, the material can be assumed to be nearly or even entirely amorphous and have constructed synthetic curves for the thermal properties based on this assumption. The general process of obtaining these results should be repeatable for any semi-crystalline material providing that the same assumptions regarding the crystallinity can be applied.

It is also possible to expand on the experimental side by utilizing other measurement techniques. Laser-Flash-Analysis (LFA) may be used as an alternative measurement method to verify the thermal conductivity measurements shown above. Flash DSC tests would allow for the measurement of cooling curves at cooling rates comparable to those in the FFF process, potentially eliminating the need for a synthetic heat capacity curve altogether.

## Figures and Tables

**Table 1:** Results of DSC analysis for all tested sample groups.

	$T_{c}$	$T_{m}$	$\chi_{c}$ Standard	$\chi_{c}$ Ref. Shifted	$\chi_{c}$ Combined
	°C	°C	%	%	%
Printed	112 ± 0.4	236 ± 0.2	11.36 ± 0.36	8.26 ± 0.33	4.58 ± 0.76
Quenched	111 ± 1.1	236 ± 0.3	10.11 ± 0.26	7.02 ± 0.34	1.58 ± 1.14
IM Near	106 ± 1.0	238 ± 0.3	8.38 ± 0.18	5.93 ± 0.38	−3.00 ± 0.90
IM Far	107 ± 2.1	239 ± 1.7	7.61 ± 2.73	5.50 ± 3.07	−2.70 ± 5.22
IM Gauge	106 ± 0.7	237 ± 1.1	10.73 ± 1.73	9.11 ± 1.69	4.26 ± 5.57
IM All	106 ± 1.4	238 ± 1.2	8.91 ± 2.15	6.85 ± 2.45	−0.48 ± 5.24

**Table 2:** Synthetic $c_{p}$ curve parameters.

	$T_{g}$ Onset	$T_{g}$ Endset	$m_{i}$	$t_{i}$	a	b	c	d
	°C	°C	J/g °C^2^	J/g °C	J/g °C	J/g °C	°C	°C
Pre $T_{g}$	64.04	76.92	0.0075	1.0810				
Simul $T_{g}$	64.04	76.92			1.5612	0.3380	70.4822	3.8
Post $T_{g}$	64.04	76.92	0.0031	1.6578				

**Table 3:** Synthetic λ curve parameters.

	a	b	c	d
	W/m °C^2^	W/m °C^2^	°C	W/m °C
λ(θ)	4.101 × 10^−4^	−4.640 × 10^−4^	7.150 × 10^1^	2.323 × 10^−1^

**Table 4:** Synthetic ρ curve parameters.

	a	b	c	d	e	m	t
	g/cm^3^ °C	g/cm^3^ °C	1/°C	°C	g/cm^3^	g/cm^3^ °C	g/cm^3^
<140 °C	−1.9813 × 10^−4^	−3.6727 × 10^−4^	4.3138 × 10^−2^	9.3918 × 10^1^	1.1644 × 10^0^		
≥140 °C						−4.7904 × 10^−4^	1.1828 × 10^0^

## Data Availability

Further methodological details and explanations are included in the Supplementary Materials. The raw data supporting the conclusions of this article will be made available by the authors on request.

## Supplementary Materials

The following supporting information can be downloaded at: https://www.mdpi.com/article/10.3390/polym18080981/s1.
