# Supplementary material for: Temperature-Dependent Thermal Properties of Nearly Amorphous Polyamide 6
Source: Polymers (Basel). 2026 Apr 17;18(8):981. doi: 10.3390/polym18080981 (PMC13120163; doi:10.3390/polym18080981)
Supplement: Supplementary file 1 [file polymers-18-00981-s001.zip › polymers-4181538-supplementary.pdf]

Supplementary Materials: Temperature-Dependent Thermal Properties of Nearly Amorphous Polyamide 6

Julian Klingenbeck, Alexander Lion and Michael Johlitz

1. Reference enthalpy correction procedure

In the following, a detailed explanation of the reference enthalpy correction procedure utilized in section 3.1.1 for the method following Millot et al. [1] is given. As discussed above, the reference enthalpy taken from literature for a 100 % crystalline sample (230 J/g at 533 K) is corrected by employing Kirchhoff's law:

$$\Delta H_f(T_2) - \Delta H_f(T_1) = \int \Delta c_p \, dT \tag{S1}$$

With the change in heat capacity Δc_p given as:

$$\Delta c_p = c_{p,l}(T) - c_{p,s}(T) \tag{S2}$$

In order to utilize these equations, the heat capacity of the amorphous (liquid) and crystalline (solid) phase of the material are required. Following the example of Millot et al., we use the data from Gaur et al. [2], who provide experimental tabular data for the amorphous (liquid) and crystalline (solid) heat capacities of several polymers. Based on the provided tabular data, the curves depicted in Fig. S1 were created. The molar mass needed to convert the provided heat capacity to specific heat capacity was calculated to be 113.16 [g/mol] according to the periodic table and the structural formula of polyamide 6.

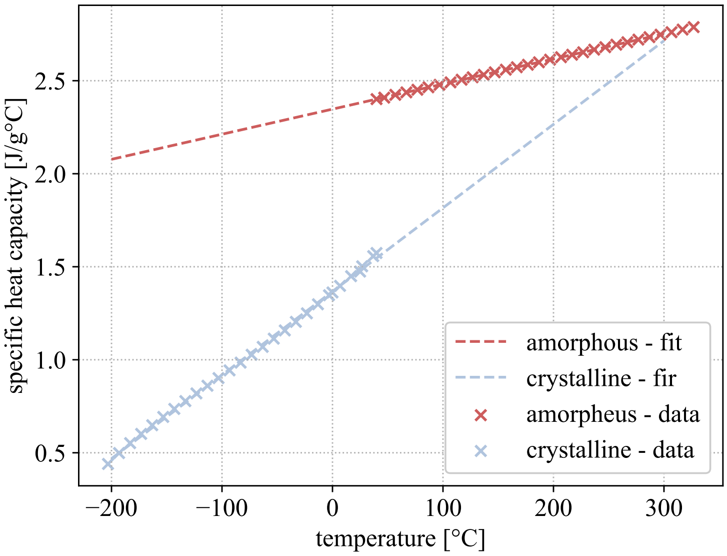

Figure S1. Reference heat capacity values for amorphous (liquid) and crystalline (solid) polyamide 6 based on Gaur et al [1983].

The data points (cross markers) represent the original data. In order to obtain the necessary data across the entire temperature range for both heat capacities two linear functions were fitted to the data sets using the polyfit function of the Python NumPy library, resulting in the following linear functions:

$$c_{p,l} = 0.001349\,T + 2.3472 \qquad \left[\frac{\text{J}}{\text{g}\,°\text{C}}\right] \tag{S3}$$

$$c_{p,s} = 0.004492\,T + 1.3672 \qquad \left[\frac{\text{J}}{\text{g}\,°\text{C}}\right] \tag{S4}$$

Using the above formulas, Δc_p can be calculated as:

$$\Delta c_p = -0.003143\,T + 0.98 \qquad \left[\frac{\text{J}}{\text{g}\,°\text{C}}\right] \tag{S5}$$

and after integration:

$$\int \Delta c_p\,dT = -0.0015715\,T^2 + 0.98\,T + c \qquad \left[\frac{\text{J}}{\text{g}}\right] \tag{S6}$$

Using the known reference melting enthalpy, we can set the left-hand side of equation (6) to 230 J/g and insert a temperature of 259.85 °C (533 K) on the right-hand side to calculate the integration constant c and arrive at:

$$\int \Delta c_p\,dT = -0.0015715\,T^2 + 0.98\,T + 81.4579 \qquad \left[\frac{\text{J}}{\text{g}}\right] \tag{S7}$$

This equation now directly gives the reference melting enthalpy, also known as heat of fusion, for polyamide 6 at a desired temperature, as depicted in Fig. S2.

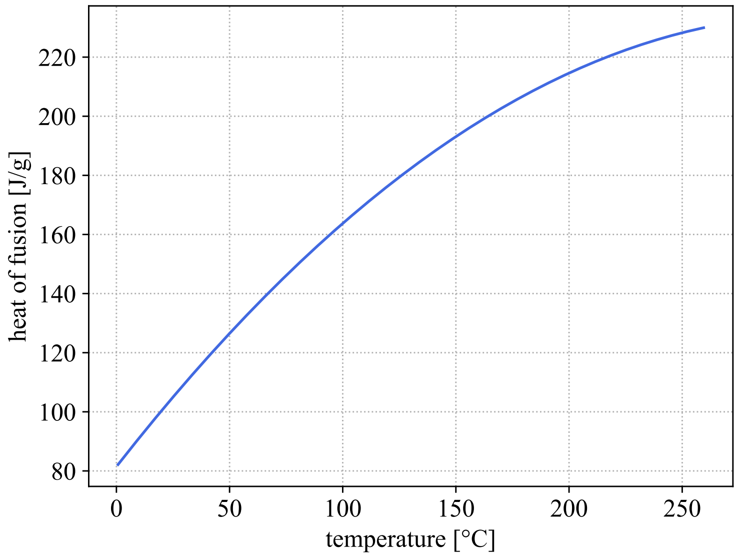

Figure S2. Reference heat of fusion for polyamide 6 based on equation (A7)

2. Tabular data for isobaric heat capacity, thermal conductivity and density of PA 6 based on equations (7) - (10) from main the main text

In the following, selected values for heat capacity, thermal conductivity, and density are given for selected temperature values based on the synthetic data curves provided above.

Table S1. Isobaric Heat Capacity - Polyamide 6

T	c_p	T	c_p
°C	J/g °C	°C	J/g °C
0	1.081	155	2.1383
5	1.119	160	2.1538
10	1.156	165	2.1693
15	1.194	170	2.1848
20	1.231	175	2.2003
25	1.269	180	2.2158
30	1.306	185	2.2313
35	1.344	190	2.2468
40	1.381	195	2.2623
45	1.419	200	2.2778
50	1.456	205	2.2933
55	1.494	210	2.3088
60	1.531	215	2.3243
65	1.568	220	2.3398
70	1.706	225	2.3553
75	1.884	230	2.3708
80	1.906	235	2.3863
85	1.921	240	2.4018
90	1.937	245	2.4173
95	1.952	250	2.4328
100	1.968	255	2.4483
105	1.983	260	2.4638
110	1.999	265	2.4793
115	2.014	270	2.4948
120	2.030	275	2.5103
125	2.045	280	2.5258
130	2.061	285	2.5413
135	2.076	290	2.5568
140	2.092	295	2.5723
145	2.107	300	2.5878
150	2.123		

Table S2. Thermal Conductivity - Polyamide 6

T	λ	T	λ
°C	W/m K	°C	W/m K
0	0.2323	155	0.2571
5	0.2344	160	0.2569
10	0.2364	165	0.2566
15	0.2385	170	0.2563
20	0.2405	175	0.2560
25	0.2426	180	0.2558
30	0.2446	185	0.2555
35	0.2467	190	0.2552
40	0.2487	195	0.2550
45	0.2508	200	0.2547
50	0.2528	205	0.2544
55	0.2549	210	0.2542
60	0.2569	215	0.2539
65	0.2590	220	0.2536
70	0.2610	225	0.2534
75	0.2614	230	0.2531
80	0.2612	235	0.2528
85	0.2609	240	0.2525
90	0.2606	245	0.2523
95	0.2604	250	0.2520
100	0.2601	255	0.2517
105	0.2598	260	0.2515
110	0.2596	265	0.2512
115	0.2593	270	0.2509
120	0.2590	275	0.2507
125	0.2587	280	0.2504
130	0.2585	285	0.2501
135	0.2582	290	0.2498
140	0.2579	295	0.2496
145	0.2577	300	0.2493
150	0.2574		

Table S3. Density - Polyamide 6

T	ρ	T	ρ
°C	g/cm^3	°C	g/cm^3
0	1.1644	155	1.1086
5	1.1634	160	1.1062
10	1.1624	165	1.1038
15	1.1614	170	1.1014
20	1.1603	175	1.0990
25	1.1592	180	1.0966
30	1.1582	185	1.0942
35	1.1570	190	1.0918
40	1.1559	195	1.0894
45	1.1547	200	1.0870
50	1.1534	205	1.0846
55	1.1520	210	1.0822
60	1.1506	215	1.0798
65	1.1491	220	1.0774
70	1.1474	225	1.0750
75	1.1457	230	1.0726
80	1.1438	235	1.0702
85	1.1417	240	1.0678
90	1.1396	245	1.0654
95	1.1374	250	1.0630
100	1.1350	255	1.0606
105	1.1326	260	1.0583
110	1.1302	265	1.0559
115	1.1278	270	1.0535
120	1.1253	275	1.0511
125	1.1229	280	1.0487
130	1.1205	285	1.0463
135	1.1181	290	1.0439
140	1.1157	295	1.0415
145	1.1133	300	1.0391
150	1.1109		

References

1. Millot, C.; Fillot, L.A.; Lame, O.; Sotta, P.; Seguela, R. Assessment of polyamide-6 crystallinity by DSC: Temperature dependence of the melting enthalpy. *J. Therm. Anal. Calorim.* **2015**, *122*, 307–314. https://doi.org/10.1007/s10973-015-4670-5.
2. Gaur, U.; Lau, S.F.; Wunderlich, B.B.; Wunderlich, B. Heat Capacity and Other Thermodynamic Properties of Linear Macromolecules. VIII. Polyesters and Polyamides. *J. Phys. Chem. Ref. Data* **1983**, *12*, 65–89. https://doi.org/10.1063/1.555678.

Disclaimer/Publisher's Note: The statements, opinions and data contained in all publications are solely those of the individual author(s) and contributor(s) and not of MDPI and/or the editor(s). MDPI and/or the editor(s) disclaim responsibility for any injury to people or property resulting from any ideas, methods, instructions or products referred to in the content.
